# Association of Mean Arterial Pressure (MAP) with Mortality in Patients with Liver Cirrhosis Awaiting Transplantation

**DOI:** 10.3390/jcm15166292

**Published:** 2026-08-14

**Authors:** Yazan Omari, Ahmad Alomari, Ismail Althunibat, Abdulmalik Saleem, Thai Hau Koo, Yara Dababneh, Diana Jomaa, James Mo, Ahmad Abdulraheem, Syed-Mohammed Jafri

**Affiliations:** 1 Henry Ford Providence Hospital, Southfield, MI 48075, USA; 2 Henry Ford Health, Detroit, MI 48202, USA

**Keywords:** mean arterial pressure, liver cirrhosis, liver transplantation, hepatorenal syndrome, waiting lists

## Abstract

**Background/Objectives:** In cirrhotic patients, guidelines generally recommend maintaining mean arterial pressure (MAP) ≥ 65 mmHg, but the prognostic impact of MAP in transplant candidates is unclear. This study aimed to evaluate the association between MAP and waitlist mortality and cirrhosis-related complications in patients listed for liver transplantation. **Methods:** We conducted a retrospective cohort study of 103 adults (age ≥ 18 years) with cirrhosis listed for liver transplantation (MELD 20–24) at a single center (2019–2023). Patients with hepatocellular carcinoma were excluded. The primary outcome was death on the transplant waitlist (*n* = 9 events). Logistic regression was used to assess the association of MAP (per 1 mmHg) with mortality. Continuous variables were compared using the t-test, and categorical variables were compared using the chi-square test; *p* < 0.05 was considered significant. **Results:** Mean MAP at listing was significantly higher in survivors than non-survivors (83.2 ± 9.4 vs. 76.9 ± 8.7 mmHg; *p* = 0.04). In logistic regression, higher MAP was associated with lower odds of waitlist death (unadjusted odds ratio [OR] per 1 mmHg increase = 0.94; 95% confidence interval [CI] 0.89–0.99; *p* = 0.041). Subgroup analysis showed a significant inverse association between MAP and hepatorenal syndrome (HRS) (OR per 1 mmHg = 0.94; 95% CI 0.89–0.98; *p* = 0.011), whereas MAP was not significantly associated with hepatic encephalopathy, ascites, or variceal bleeding (all *p* > 0.2). **Conclusions:** Among the cirrhotic patients listed for transplantation, lower MAP at baseline is associated with waitlist mortality and hepatorenal syndrome. These findings should be interpreted cautiously given the small number of events and require validation in larger cohorts.

## 1. Introduction

Liver cirrhosis is a major global health burden characterized by progressive fibrosis and hepatic dysfunction. Recent data estimate that liver cirrhosis affects approximately 2.2 million adults in the United States and is responsible for roughly 1.5 million deaths worldwide in 2019 (an 8.1% increase since 2017), which is a major contributor to morbidity and mortality in patients with liver disease [1,2]. The leading causes include alcohol-related liver disease, chronic viral hepatitis, and metabolic dysfunction-associated fatty liver disease [1,2,3]. Currently, the only definitive treatment for liver cirrhosis is transplantation. However, with the limited number of liver transplants available at a given time, monitoring and optimization of patients with liver cirrhosis through medical management are needed while patients are being evaluated for liver transplants.

Cirrhosis can remain compensated for years, but eventually portal hypertension ensues. As liver cirrhosis progresses, patients can develop hemodynamic instability due to portal hypertension and peripheral vasodilation [3]. One proposed mechanism is increased production and decreased metabolism of circulating vasodilators, which causes venous dilation, endothelial damage, and collateral pathway formation. Vasodilators include nitric oxide, eicosanoids, bile salts, adenosine, substance P, and calcitonin gene-related peptides. This hyperdynamic syndrome associated with cirrhosis unloads baroreceptors and activates vasoconstrictor systems (renin–angiotensin–aldosterone system, sympathetic nervous system, and vasopressin release) to maintain perfusion but ultimately leads to high cardiac output, low peripheral resistance, and reduced effective arterial blood volume [4]. These hemodynamic changes underlie the serious complications of advanced cirrhosis, which increase the risk of multi-organ failure, including refractory ascites, spontaneous bacterial peritonitis, portal hypertensive gastropathy, variceal hemorrhage, hepatic encephalopathy, and hepatorenal syndrome (HRS) [5]. In particular, systemic hypotension and decreased MAP have been implicated in renal hypoperfusion and the development of HRS, which complicate the management of cirrhosis and worsen patient outcomes [3,6,7].

The mean arterial pressure (MAP) is a key indicator of global circulatory perfusion. Vasoconstrictors, such as noradrenaline, midodrine, terlipressin, and octreotide, are used to raise MAP and stabilize the patient’s circulatory status [8]. In critically ill cirrhotic patients (e.g., sepsis and multi-organ failure), where maintaining adequate organ perfusion is essential, expert opinion often targets MAP ≥ 65–70 mmHg to ensure adequate renal and cerebral blood flow, especially when they experience additional organ failure [6]. However, in routine outpatient or pre-transplant care, the significance of low MAP is less well-defined. Existing studies suggest that cirrhotic patients with chronically low MAP are at risk for complications. For example, Cullaro et al. identified outpatient MAP trajectories as predictors of acute kidney injury and waitlist mortality [9]. Beta-blocker use in cirrhosis also exemplifies this interplay: nonselective beta-blockers (NSBBs) reduce portal pressure but can exacerbate hypotension, potentially precipitating renal dysfunction if the MAP drops below ~65 mmHg [10]. Despite these insights, there are limited data specifically on how resting MAP at transplant listing impacts subsequent survival on the waiting list. Given the vital role of MAP in managing these patients, monitoring it can help guide clinical interventions and determine the prognosis for patients awaiting liver transplantation.

Despite advancements in therapy, mortality rates remain high among patients on the waiting list for transplantation, which is the only definitive treatment for liver cirrhosis. At a given time, only about 6000 out of the 13,000 to 15,000 patients listed receive a transplant, and approximately 2000 patients die while waiting for one [11]. Risk stratification for transplant candidates traditionally relies on MELD scores and listed complications, but many patients with similar MELD still have heterogeneous outcomes. This study aimed to investigate the relationship between MAP and mortality in patients with liver cirrhosis who were listed for liver transplantation. We sought to offer insights into how monitoring and adjusting MAP might improve outcomes for these patients. We hypothesized that a lower MAP at listing, reflecting more severe circulatory dysfunction, would be associated with waitlist mortality in patients with cirrhosis. Here, we analyzed our center’s experience to assess the association between baseline MAP and death while awaiting liver transplantation, adjusting for other clinical factors.

## 2. Materials and Methods

### 2.1. Study Design and Population

We conducted a retrospective cohort study of adult patients (aged ≥ 18 years) with liver cirrhosis who were listed for liver transplantation at a quaternary care center between January 2019 and December 2023. Ethical approval was obtained from the Institutional Review Board (IRB; protocol code 17104, date of approval 21 June 2024). This retrospective study was exempt from informed consent. This study was conducted in accordance with the ethical standards of the Institutional Research Committee and the 1964 Declaration of Helsinki and its later amendments. This study followed the Strengthening the Reporting of Observational Studies in Epidemiology (STROBE) guidelines.

### 2.2. Inclusion and Exclusion Criteria

Patients who were 18 years old or above with a diagnosis of liver cirrhosis by imaging or biopsy, registered on the transplant waitlist with a laboratory Model for End-Stage Liver Disease (MELD) score of 20 to 24, and available baseline hemodynamic measurements were included in this study. We selected the MELD 20–24 range to focus on patients with moderate disease severity and reduce heterogeneity. Patients listed for transplantation primarily for hepatocellular carcinoma (HCC) were excluded because their inclusion could confound the outcomes of interest due to the difference in their prognosis and management protocols.

### 2.3. Data Collection

The data for this study were sourced from the electronic medical records of adults who had been diagnosed with liver cirrhosis and were placed on the list for liver transplantation. The demographic details gathered included age, sex, and race, along with the clinical characteristics in the form of mean arterial pressure at listing time and other cirrhosis-associated complications such as hepatorenal syndrome (HRS), hepatic encephalopathy (HE), ascites, and variceal bleeding documented in the medical record at any point from the time of listing through to the end of follow-up (i.e., death, transplantation, or study end). Medications of interest recorded at listing included nonselective beta-blockers (propranolol, nadolol, and carvedilol), midodrine, diuretics (spironolactone), and ACE inhibitors/ARBs. The clinical outcome was death on the transplant waiting list. Patients who were transplanted or still alive by the study end were considered survivors for this analysis; those removed from the list for other reasons (e.g., improvement, transfer) were censored or excluded.

### 2.4. Statistical Analysis

The data were analyzed using SPSS version 23.0. We first described the baseline characteristics using means (±SD) or medians (IQR) for continuous variables and frequencies (%) for categorical variables. To compare survivors and non-survivors, we used *t*-tests or Mann–Whitney tests for continuous variables (depending on normality) and chi-square or Fisher’s exact tests for categorical variables.

The association between MAP and odds of waitlist death was assessed using logistic regression. MAP was modeled as a continuous variable (per 1 mmHg increment). We first ran unadjusted (univariate) logistic regression and then constructed a multivariable model adjusting for potential confounders: specifically, the presence of each cirrhosis complication (HRS, HE, ascites, variceal bleeding) and the use of vasoactive medications. Variables were selected for the model based on clinical relevance and univariate associations with the outcome (*p* < 0.10). We checked for model fit and collinearity. Results are presented as odds ratios (ORs) with 95% confidence intervals (CIs). A *p*-value < 0.05 (two-tailed) was considered statistically significant.

Given the low number of outcome events (n = 9 deaths), the number of covariates that can be reliably included in multivariable models is limited. The traditional guideline of approximately 10 events per predictor variable (EPV) cannot be met with this event count [12,13]. Therefore, the unadjusted analysis is presented as the primary result, and the adjusted analysis should be interpreted as exploratory.

To illustrate differences in MAP between groups, we compared the mean MAP values between survivors and non-survivors using a two-sample t-test. We also constructed predicted probability curves of death versus MAP using the logistic model to visualize the risk thresholds. The subgroup analyses examined the associations of MAP with each complication (e.g., HRS) using separate logistic regressions.

## 3. Results

### 3.1. Demographic Characteristics

The baseline demographic characteristics of the study population are presented in Table 1. A total of 103 patients met the inclusion criteria. During the study period, 9 patients (8.7%) died on the waitlist. The cohort was 63.1% female and 36.9% male. The mean age of the population was 57.6 ± 11.4 years, with ages ranging from 25 to 77 years. Most individuals were Caucasian (79.6%), followed by African American (11.7%), Hispanic (3.9%), Middle Eastern (1.9%), and other or unknown (2.9%).

### 3.2. Association Between MAP and Waitlist Mortality

**Figure 1:**
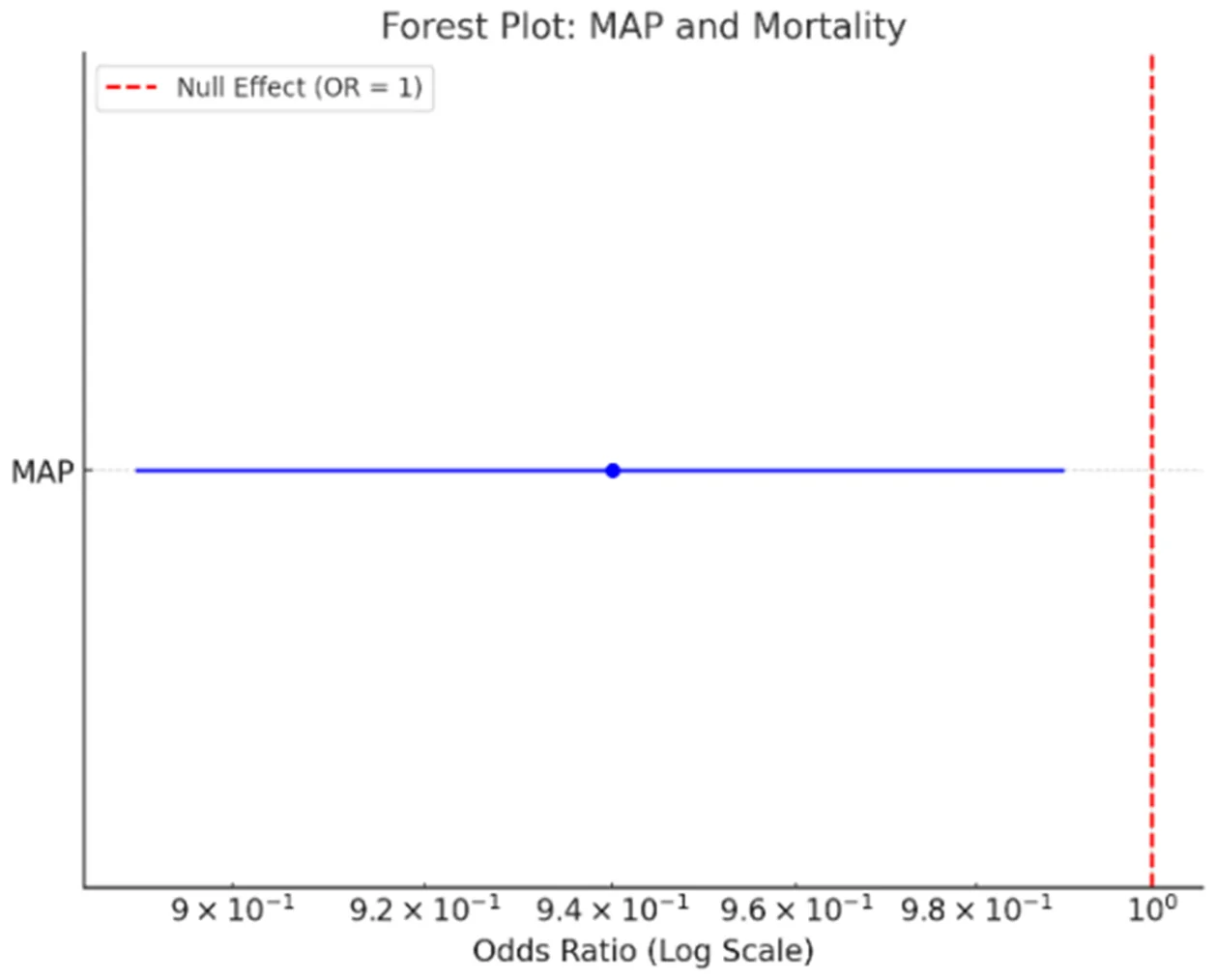
MAP in survivors compared with non-survivors. The bar chart presents the mean MAP for survivors (83.2 mmHg) and non-survivors (76.9 mmHg). Error bars indicate standard deviation within each group.

Mean arterial pressure at listing was significantly higher in survivors (83.2 ± 9.4 mmHg) than in those who died (76.9 ± 8.7 mmHg; *p* = 0.04; Figure 1).

In the unadjusted logistic regression (primary analysis), each 1 mmHg increase in MAP corresponded to OR = 0.94 for death (95% CI 0.89–0.99; *p* = 0.041), demonstrating that higher MAP was associated with lower odds of death. In an exploratory multivariable model adjusted for cirrhosis complications and medications, the association remained virtually unchanged (adjusted OR per 1 mmHg = 0.94; 95% CI 0.89–0.99; *p* = 0.041). However, given the low number of events (n = 9) relative to the number of covariates in the adjusted model, the multivariable results should be considered exploratory, as the model exceeds recommended events-per-variable thresholds [12,13].

**Figure 2:**
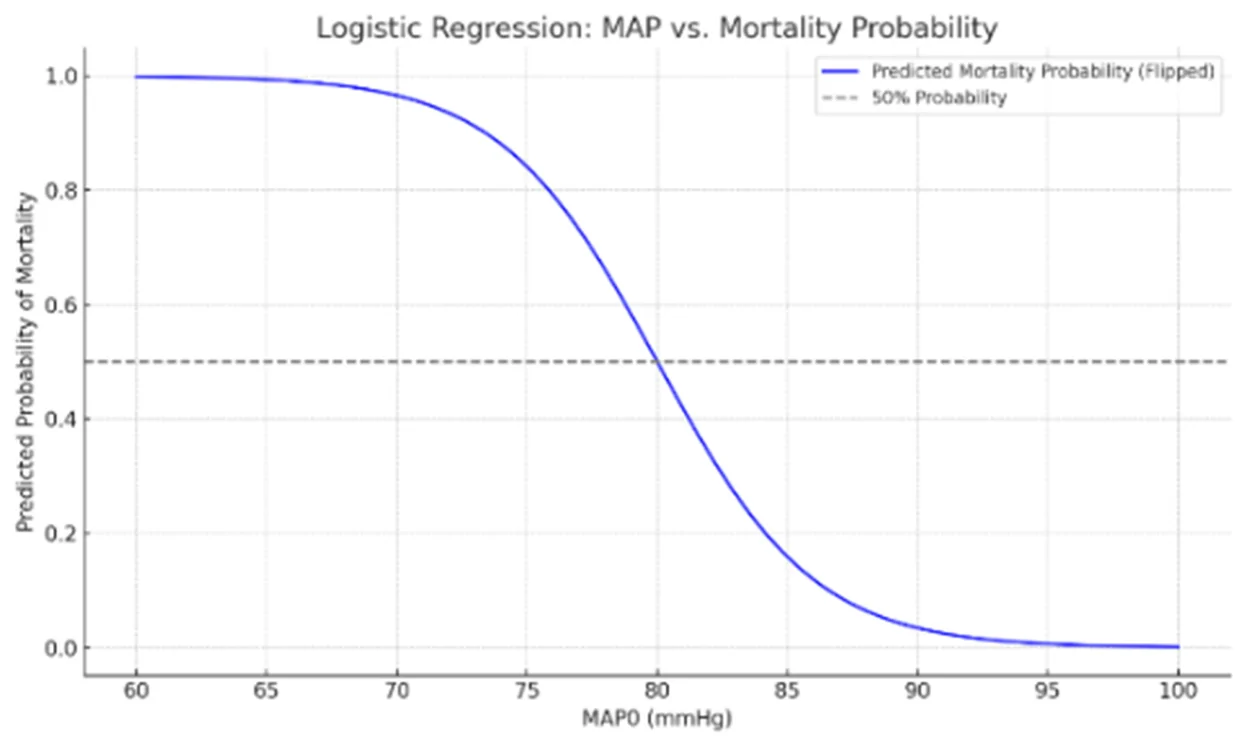
Logistic regression predicted probability of mortality as a function of MAP. The logistic curve shows how the probability of mortality decreases with increasing MAP. A dashed line indicates a threshold of 50% probability.

The logistic regression model showed that the predicted probability of mortality decreased with increasing MAP levels (Figure 2). The predicted probability curve showed a steep decrease in mortality risk as MAP rose from very low values, plateauing at higher MAP.

No other baseline demographic or laboratory variables (including MELD and serum creatinine) differed significantly between survivors and non-survivors in univariate testing.

### 3.3. Subgroup Analysis: MAP and Cirrhosis-Related Complications

Subgroup analysis examined the association between baseline MAP at listing and cirrhosis-related complications documented during the waitlist period (Table 2). These complications were not mutually exclusive and were ascertained from the time of listing through death, transplantation, or study end (Table 2). The strongest finding was for hepatorenal syndrome: each 1 mmHg increase in MAP was associated with 6% lower odds of HRS (OR = 0.94; 95% CI 0.89–0.98; *p* = 0.011). In contrast, MAP showed no significant relationship with hepatic encephalopathy (OR = 0.97; 95% CI 0.93–1.02; *p* = 0.247), ascites (OR = 1.00; 95% CI 0.95–1.05; *p* = 0.847), or variceal bleeding (OR = 1.02; 95% CI 0.95–1.09; *p* = 0.567).

## 4. Discussion

Our study found that lower MAP at the time of transplant listing was associated with increased mortality among patients listed for liver transplantation due to cirrhosis. Survivors exhibited significantly higher baseline MAP than non-survivors (83.2 vs. 76.9 mmHg). In unadjusted logistic regression, each 1 mmHg increase in MAP was associated with approximately 6% lower odds of waitlist death (OR 0.94; 95% CI 0.89–0.99). Additionally, a similar relationship was observed between MAP and the incidence of HRS: patients with lower MAP had higher odds of HRS. In contrast, MAP was not significantly associated with HE, ascites, or variceal bleeding. These findings suggest a potential role for MAP monitoring in the risk stratification of transplant candidates.

Our findings align closely with emerging evidence that systemic hypotension in cirrhosis is associated with poor outcomes. Cullaro et al. recently reported that lower outpatient MAP trajectories predicted both acute kidney injury and waitlist mortality in a large multicenter cirrhosis cohort of 1786 patients [9]. In their adjusted models, each 10 mmHg increment in MAP corresponded to an approximately 11% lower hazard of waitlist death, and an MAP threshold of 82 mmHg was identified as most associated with outcomes [9]. Similarly, Badura et al. (2023) highlighted in their review that systemic hypotension is a hallmark of decompensated cirrhosis and a precursor to HRS, supporting the notion that decreased MAP is associated with HRS [14]. Smith et al. (2024) demonstrated an association between MAP 65 mmHg and short-term mortality in patients with acute cirrhosis complications, which complements our focus on longer-term outcomes and the relevance of MAP thresholds [15]. Intensive care studies have shown that persistent hypotension dramatically increases short-term mortality in patients with cirrhosis. Patidar et al. found in a 273-patient ICU cohort that a time-weighted average MAP below 65–70 mmHg was strongly linked to higher ICU mortality [6]. Importantly, even after controlling for illness severity (MELD-Na, ventilation, vasopressors, etc.), maintaining MAP > 65 mmHg was associated with better survival [6]. Our study extends these insights to the pre-transplant outpatient setting, suggesting that MAP may be a valuable prognostic marker even before critical illness ensues.

Physiologically, these observations are consistent with the pathophysiology of advanced cirrhosis. Cirrhosis-induced vasodilation reduces effective arterial blood volume and renal perfusion [5,16]. When MAP falls, renal blood flow (which is already tenuous due to cirrhotic vasoconstriction) declines further, precipitating HRS. Indeed, we found that higher MAP was significantly associated with a lower risk of HRS. This is consistent with data from HBV-related acute-on-chronic liver failure (ACLF), where Zheng et al. showed that a drop in MAP ≥ 9.5 mmHg was an independent predictor of HRS and short-term mortality [7]. In cirrhotic patients with spontaneous bacterial peritonitis or HRS, restoration of MAP via vasoconstrictors (e.g., terlipressin or norepinephrine) is known to improve renal outcomes, further highlighting the key role of arterial pressure in renal perfusion. Nadim et al. explored therapeutic interventions, such as midodrine and other vasoconstrictors, to optimize MAP in cirrhosis [17]. These interventions, coupled with our findings, suggest that proactive hemodynamic management could potentially enhance survival outcomes in liver transplant candidates. Of note, the exclusion of patients listed primarily for hepatocellular carcinoma limits generalizability, as HCC represents an increasingly common indication for liver transplantation. Future multicenter studies should include HCC-listed patients to determine whether the MAP–mortality association extends to this population.

It is important to note that HRS may represent a mediator on the causal pathway between low MAP and death (i.e., low MAP → renal hypoperfusion → HRS → death) rather than an independent confounder. Including HRS as a covariate in the mortality model could attenuate the total effect of MAP on mortality and introduce collider bias. For this reason, we present HRS as a secondary outcome rather than a covariate in the primary mortality analysis, and the significant association between MAP and HRS (OR 0.94 per mmHg; *p* = 0.011) supports this mechanistic pathway.

Our results also have implications for established therapies. NSBBs are standard variceal prophylaxis, but they can exacerbate hypotension. Prior work introduced the concept of a “therapeutic window” for NSBBs in advanced cirrhosis; patients with refractory ascites and MAP 65 mmHg had higher renal impairment on NSBBs than those with a higher MAP [10]. In our context, this suggests that cautious use or dose reduction in NSBBs is warranted in hypotensive transplant candidates. Our cohort included patients on beta-blockers without a reported detrimental mortality impact; however, future analyses could examine whether MAP modifies the benefit–risk of NSBBs.

From a clinical perspective, our study underscores MAP as a simple and routinely available vital sign with potential prognostic value. Unlike complex biomarkers, MAP requires no special testing and can be used to guide management. Candidates with chronically low MAP may benefit from closer monitoring, adjustment of diuretics, or even empiric hemodynamic support. Therapies such as midodrine (an oral alpha-agonist) have been tested in cirrhosis to increase MAP. A randomized trial (MACHT study) found that midodrine plus albumin did not significantly reduce death or complications in waitlisted cirrhotic patients, although it modestly suppressed renin activity [18]. The TARGET-C trial found no overall mortality difference between high (85 mmHg) and low (60 mmHg) MAP targets in septic cirrhotic patients; however, it did show better AKI reversal with higher MAP [19]. Whether similar nuances apply to ambulatory patients requires further investigation. An important area for future investigation is whether baseline MAP at listing predicts early post-transplant outcomes, such as ICU length of stay and perioperative complications. Such data were not collected in the present study but could further support the clinical utility of MAP monitoring in transplant candidates.

While our findings highlight MAP’s potential predictive value, they do not establish causation. A lower MAP likely reflects more advanced circulatory dysfunction, which itself causes organ failure. Nonetheless, identifying MAP as a potentially modifiable factor opens avenues for investigation; interventions to prevent or correct hypotension could plausibly extend survival.

## 5. Limitations

This study has several important limitations that warrant careful consideration.

First, the most critical limitation is the small number of outcome events (n = 9 deaths among 103 patients). This severely limits statistical power and the number of covariates that can be reliably included in multivariable models. The traditional guideline of approximately 10 events per predictor variable (EPV) cannot be met with this event count, even with a parsimonious model [12,13]. The adjusted multivariable model included more covariates than the event count could support, and therefore the adjusted results should be considered exploratory. The point estimate from standard logistic regression (OR 0.94 per 1 mmHg) may be inflated relative to the true effect, as suggested by comparison with the larger Cullaro et al. study (n = 1786), which found a more modest association (adjusted HR 0.89 per 10 mmHg) [9]. Future studies with larger sample sizes should consider Firth’s penalized logistic regression to reduce small-sample bias in coefficient estimates when events are rare [20].

Second, we used logistic regression rather than time-to-event analysis. Logistic regression does not account for variable follow-up time or censoring. While follow-up durations were broadly similar across patients, Cox proportional hazards regression would be more appropriate for time-to-event data. Additionally, transplantation represents a competing event that may preclude the observation of waitlist death. In the primary analysis, transplanted patients were classified as survivors, but patients who undergo transplantation may differ systematically from those who remain on the waitlist. A Fine–Gray competing risks regression treating death as the event of interest and transplantation as the competing event would provide a more rigorous analytical framework and should be considered in future studies.

Third, the retrospective single-center design introduces the potential for selection bias and limits generalizability. Differences in patient populations, clinical management, and transplant listing practices across institutions may influence both MAP measurements and outcomes.

Fourth, MAP was assessed at a single time point at the time of transplant listing. This approach does not capture longitudinal changes in hemodynamics or the effects of therapeutic interventions over time. Prior studies have suggested that dynamic changes in MAP, including MAP trajectories, may provide additional prognostic information beyond single measurements [9].

Fifth, residual confounding cannot be excluded. Although medications and complications were recorded, unmeasured factors such as systemic inflammation, nutritional status, cardiac output, and severity of portal hypertension may have influenced the observed associations [5]. Additionally, the baseline characteristics table does not include stratification by outcome for all clinical variables (etiology, laboratory values, medications), which limits the ability to fully assess confounding.

Sixth, the etiology of cirrhosis was not systematically reported in the results, and future studies should characterize the distribution of etiologies and examine whether MAP associations differ by underlying cause of liver disease.

Finally, the exclusion of patients with hepatocellular carcinoma and restriction to a specific MELD range (20–24) enhance internal validity but may limit external applicability. These findings may not be generalizable to patients with different disease severity or alternative indications for transplantation.

## 6. Conclusions

In this single-center retrospective study, lower mean arterial pressure at the time of liver transplant listing was associated with increased waitlist mortality and hepatorenal syndrome in patients with cirrhosis (MELD 20–24). MAP represents a simple and readily available clinical parameter that may aid in the risk stratification of transplant candidates. However, these findings are limited by the small number of events (n = 9) and the constraints of logistic regression in this setting, and should be interpreted as hypothesis-generating. Prospective multicenter studies with larger sample sizes, time-to-event methodology, and competing risks analysis are needed to validate these results and to determine whether targeted interventions aimed at optimizing MAP can improve clinical outcomes in this high-risk population.

## Figures and Tables

**Table 1:** Baseline demographic and clinical characteristics of the study population, stratified by waitlist outcome.

Characteristic	Overall (N = 103)	Survivors (n = 94)	Non-Survivors (n = 9)	*p*-Value
Age, years, mean ± SD	57.6 ± 11.4	†	†	NS †
Age range, years	25–77	—	—	—
Female sex, n (%)	65 (63.1)	†	†	NS †
**Ethnicity, n (%)**				NS †
Caucasian	82 (79.6)	†	†	
African American	12 (11.7)	†	†	
Hispanic	4 (3.9)	†	†	
Middle Eastern	2 (1.9)	†	†	
Other/Unknown	3 (2.9)	†	†	
**Clinical parameters**				
MAP, mmHg, mean ± SD	82.6 ± 9.4	83.2 ± 9.4	76.9 ± 8.7	0.04
MELD score	†	†	†	NS †
Serum creatinine	†	†	†	NS †
**Medications at listing, n (%)**				
Nonselective beta-blockers	†	†	†	NS †
Midodrine	†	†	†	NS †
Diuretics (spironolactone)	†	†	†	NS †
ACE inhibitors/ARBs	†	†	†	NS †
**Complications during waitlist, n (%)**				
Hepatorenal syndrome	†	†	†	—
Hepatic encephalopathy	†	†	†	—
Ascites	†	†	†	—
Variceal bleeding	†	†	†	—

† Group-level stratified data are not available for reporting due to closure of the study database; however, univariate testing confirmed no statistically significant differences between survivors and non-survivors for any variable other than MAP (all *p* > 0.05). NS = not significant. The overall MAP was calculated as the weighted mean of survivor and non-survivor values.

**Table 2:** Association between baseline map at listing and cirrhosis-related complications during the waitlist period (logistic regression, per 1 mmHg increase in MAP).

Analysis	Events, n (%)	Odds Ratio (OR)	95% CI (Lower)	95% CI (Upper)	*p*-Value
MAP and HE	†	0.97	0.93	1.02	0.247
MAP and HRS	†	0.94	0.89	0.98	0.011
MAP and Ascites	†	1.00	0.95	1.05	0.847
MAP and Variceal Bleeding	†	1.02	0.95	1.09	0.567

† Event counts for each complication are not available for reporting due to closure of the study database. Each row represents a separate logistic regression with the respective complication as the dependent variable and MAP (per 1 mmHg) as the independent variable. Complications were ascertained from the time of listing through death, transplantation, or study end. Abbreviations: MAP, mean arterial pressure; HE, hepatic encephalopathy; HRS, hepatorenal syndrome; CI, confidence interval.

## Data Availability

Summary-level data supporting the findings of this study are presented within the paper. Individual-level data were obtained from institutional medical records and are not publicly available due to patient privacy restrictions and closure of the study database.

## Abbreviations

MAP: Mean Arterial Pressure; MELD: Model for End-Stage Liver Disease; HRS: Hepatorenal Syndrome; HE: Hepatic Encephalopathy; HCC: Hepatocellular Carcinoma; NSBB: Nonselective Beta-Blocker; MASH: Metabolic Dysfunction-Associated Steatohepatitis; ACLF: Acute-on-Chronic Liver Failure; AKI: Acute Kidney Injury; SBP: Spontaneous Bacterial Peritonitis; ACE: Angiotensin-Converting Enzyme; ARB: Angiotensin Receptor Blocker; OR: Odds Ratio; HR: Hazard Ratio; CI: Confidence Interval; EPV: Events Per Variable; IQR: Interquartile Range; IRB: Institutional Review Board; STROBE: Strengthening the Reporting of Observational Studies in Epidemiology.
